# Early catastrophic failure of Birmingham acetabular dysplasia cup in revision arthroplasty: a case report

**DOI:** 10.4076/1757-1626-2-8716

**Published:** 2009-07-30

**Authors:** Manjunath Ramappa, Andrew Port

**Affiliations:** Department Of Orthopaedics, James Cook University HospitalMiddlesbrough, TS4 3BWUK

## Abstract

**Introduction:**

Revision acetabulum arthroplasty is one of the common procedures, which has been on the rise recently. Many implants are available in the market with variable results. Aseptic loosening is the most common indication for revision acetabulum arthroplasty. Birmingham dysplasia cup has been used occasionally in these complex procedures. We know that these implants have provided good results in resurfacing dysplastic hips with bone loss. Literature review failed to answer whether these implants were as effective in revision acetabulum arthroplasty.

**Case presentation:**

We herby, present a case of revision acetabulum arthroplasty performed with Birmingham dysplasia acetabular cup, which unfortunately failed within a period of eight months. Surgical technique appeared to be adequate. On further analysis, significant differences were identified between dysplastic hips with bone loss and revision hips with bone loss.

**Conclusion:**

Therefore results obtained with dysplasia cups in resurfacing dysplastic hips does not seem to be applicable to revision hip arthroplasty. Hence these cups should be restricted to primary arthroplasty.

## Introduction

Revision acetabulum arthroplasty presents a challenge for arthroplasty surgeons. Over the years, choice of implants has constantly changed, in the quest for an ideal implant. Cementless acetabular components seem to be a favourable option at present. Its survival depends on its design, initial stability, any osteoconductive or osteoinductive properties and available host tissue.

Many strategies have been utilised for stabilisation to improve long term outcome. They include medialisation, proximal positioning of the hip centre, bone grafting, supplementary screw fixation, metal mesh or reinforcement rings, capture cups and different combinations of these techniques.

Birmingham acetabular dysplasia cup is one such implant, which has occasionally been utilised in revision surgery. It has provided good results in hip resurfacing in dysplastic hips with severe bone loss [1] and this seems to have created hope for utilisation in revision surgery. It consists of an uncemented hydroxyappatite coated porous surface with two offset threaded lugs for supplementary neutralisation screws. The acetabular component and the screws combine into one solid composite 3 dimensional construct, where the stability in any plane is a function of the area circumscribed by the component and the screws as a whole [1]. It has been utilised effectively in dysplastic hips with bone defects. But does it provide similar effect when used in revision arthroplasty was the reason behind this report.

## Case presentation

**Figure 1.**
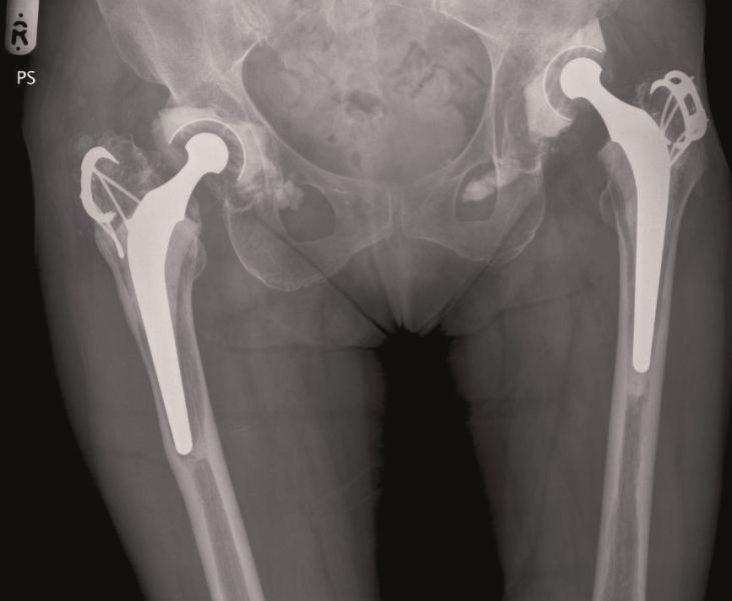
Pre operative radiograph showing loosening of left charnley total hip replacement, performed 20 years ago.

**Figure 2.**
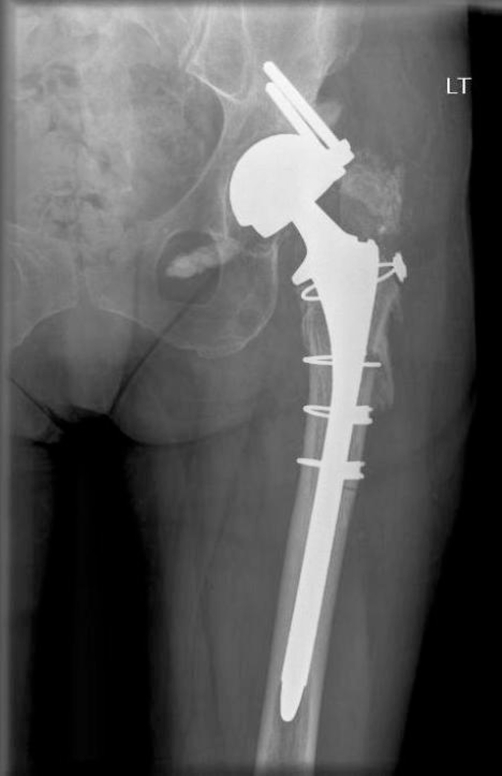
Initial postoperative radiograph showing a minimal interference gap between the acetabular cup and bone. Cup and screw positions are satisfactory.

**Figure 3.**
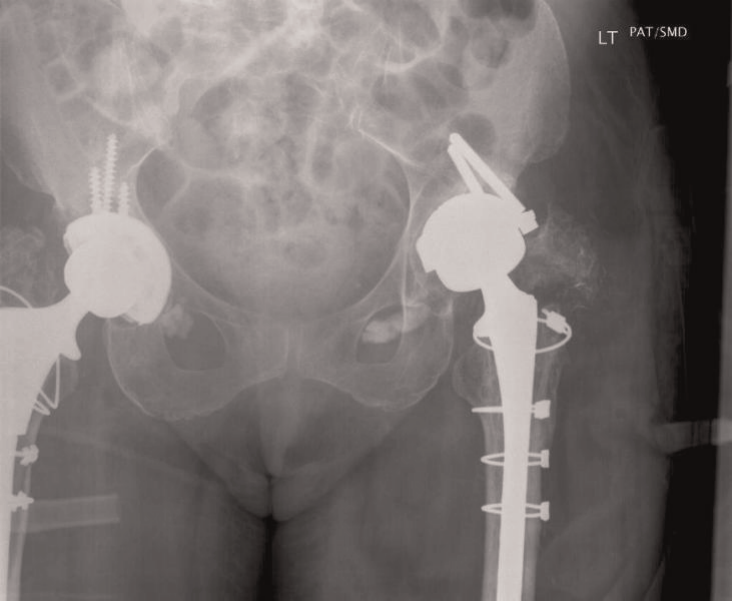
Radiograph at 8^th^ month postoperative period showing broken acetabular screws.

**Figure 4.**
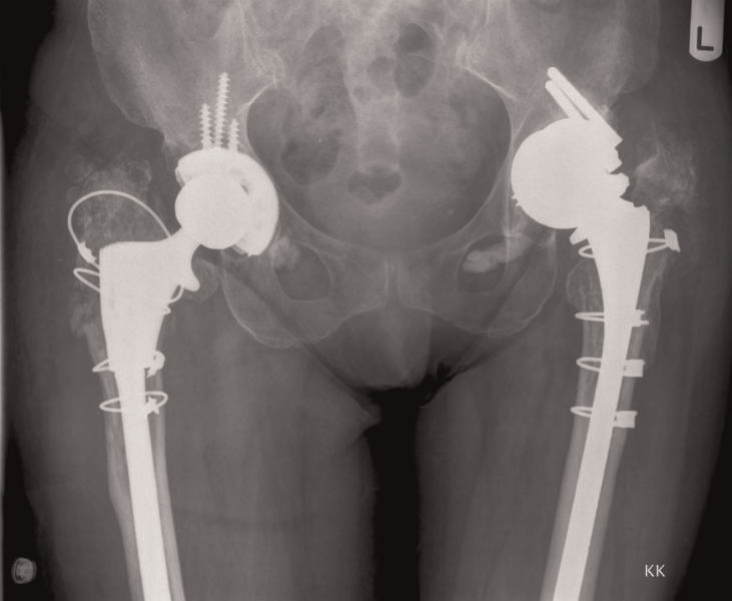
Radiograph at 12^th^ month postoperative period showing a posterior inferior migration of acetabular cup in AP view.

**Figure 5.**
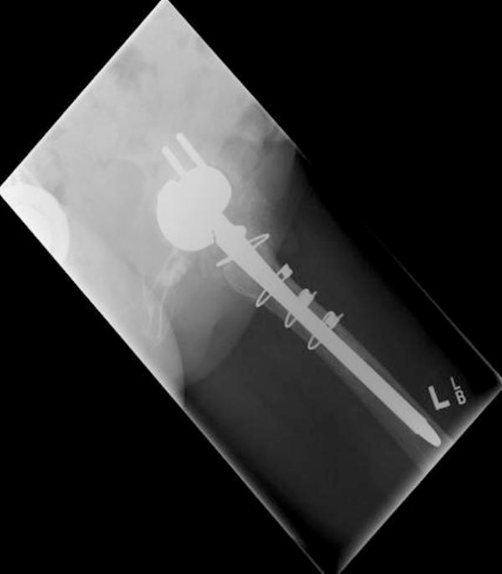
Radiograph at 12^th^ month postoperative period showing a posterior inferior migration of acetabular cup in Lateral view.

A 71 year old Caucasian lady of British origin, presented with aseptic loosening of left charnley total hip replacement, performed 20 years ago (Figure 1). Radiography revealed acetabular bone stock deficiency in the superolateral and inferior quadrants. She underwent Left revision total hip replacement with a Birmingham resurfacing dysplasia cup (HA coated size 52 mm) and femoral bone allograft in superolateral compartment, Echelon revision collared stem (190 mm) and calcar substitute, with head size measuring 42 mm. Acetabular cup was anchored with 2 suprolateral screws. Postoperative recovery was uneventful. Initial postoperative X-ray (Figure 2) showed a minimal interference gap between the acetabular cup and bone. Cup and screw positions were satisfactory. Initial mobilisation was with 2 elbow crutches for 6 weeks, 1 elbow crutch for another 6 weeks and finally onto 1 stick. Patient’s recovery was satisfactory at 6 weeks, 3 and 6 months follow up. At 8 months follow up she complained of worsening left hip pain especially with mobilisation. X-ray (Figure 3) confirmed broken acetabular screws with minimal migration of cup. Sepsis was ruled out after thorough clinical, radiological, biochemical and microbiological investigations. At 12 months there was a progressive posterior inferior migration of acetabular cup (Figures 4, 5).

## Discussion

One of the major causes for aseptic loosening in the cementless acetabular cup is its insufficient initial stability [2]. This in turn leads to fibrous tissue interposition at the metal bone interface and subsequent mechanical fatigue, due to loss of osseointegration. Birmingham dysplasia cup was produced for utilisation in dysplastic hips. Uncemented HA coated Birmingham dysplasia acetabular cups rely initially on a stable interference fit, maintained by fixation of peripheral screws, followed by biologic fixation. They have provided good results in resurfacing dysplastic hips with extensive bone loss [1].

But is primary arthroplasty in a dysplastic acetabulum with bone stock deficiency equivalent to revising an acetabular component with bone stock deficiency and aseptic loosening. If we considered patients with dysplastic hips to be in group A and patients with aseptic failure of acetabular cups to be in group B, a comparison of these two groups would identify the suitability of this dysplasia cup in the two groups.

Dysplastic patients (group A) are usually young and active in contrary to the patients in the revision group (group B) who are more often in the elderly population. Postoperative rehabilitation is much faster and easier in younger patients. Subsequently the complication rates are much lower in younger patients.

Osteoporosis, especially post menopausal, is a common finding in group B, which is a relative contraindication for resurfacing with Birmingham dysplasia cup, both due to risk of neck fracture and acetabular loosening due to poor bone quality (B).

The use of any revision acetabular component must optimize the use of whatever bone remains after primary hardware is removed. Variation in the bony and soft tissue anatomy, especially soft tissue contractures and muscle weakness in the two groups can make comparisons difficult.

Though acetabular bone deficiency can be seen in both groups, in group A there is enough sclerotic bone in the false acetabulum for the two neutralisation screws. The screws obtain good purchase in sclerotic bone of false acetabulum, rather than osteopenic bone of in the acetabular roof. (A)

Whereas in group B, aseptic loosening and biological effects of wear debris provoke peri prosthetic osteolysis and loosening of the implant. Sclerotic bone is more often than not, inadequate in this group.

In group A, as the first screw advances through the lug and reaches the prepared screw hole on the bony surface, further advancement should be very meticulous to gain initial purchase without disengaging the acetabular component from its bed. After initial purchase, the screw advances without difficulty and tightens the cup against the bone towards the end by locking. Once this screw is inserted the second screw can be inserted with relative ease. Whereas in group B, initial purchase, which is so essential can be difficult due to lack of sclerotic bone. Therefore cup has the possibility of relatively more micromovement during insertion of both first and second screws. If stable interface fit between the acetabulum cup and bone is not achieved, screws can act as potential pivot points leading to an increased micromotion which will eventually smoothen the cup surface. Smooth surface HA coated cups are known [3,4] to be associated with increased rate of aseptic loosening due to osteolysis and unstable fixation. So the acetabular component now relies heavily on the screws for positional stability. During weight transmission this can also cause differential movements between the screw, flange and cup. This not only causes fatigue failure of the screw but also loss of biologic fixation.

Excess removal of acetabular bone is usually not a problem when these cups are used in group A [5]. In revision surgery (group B), poor bone quality and bone loss is a common finding [6]. Bone loss during cup insertion or reaming [7] could further compromise cup fixation. We know that it is difficult to estimate accurate bone loss preoperatively by radiographs. Also, bone allograft has influence on failure rates of uncemented sockets [8] in the revision setting.

Theoretically, screw fixation can close any gap created by non congrous reaming [9]. Eccentric drill holes can theoretically lift the cup out of its bony bed during screw insertion, especially in revision surgery [2]. The ilium provides the least amount of support to immediate acetabular fixation, while the pubis (anterior column) and ischium (posterior column) provide more stability [10]. Eccentric screws in the ilium, in acetabuar cups with insufficient initial stability, like in group B, can therefore fail to decrease motion at the pubis or ischium significantly [10]. This can lead to fatigue failure.

In addition, locking screws will not provide compression to decrease any persistent gap between the acetabular cup=and its bed. Therefore, it is obvious that dysplastic acetabulum with bone deficiency is a completely different problem to tackle with, as compared to a revision acetabulum. Hence, good results obtained from Birmingham dysplasia cups in resurfacing dysplastic hips with severe acetabular insufficiency may not indicate its suitability for use in revision acetabulum arthroplasty.

## Conclusion

Birmingham acetabular dysplasia cup does not seem to be as forgiving a tool in revision hip arthroplasty, as compared to dysplastic hips with bone loss. Obtaining stable initial purchase during screw insertion can be difficult in revision acetabulum arthroplasty. HA coating and screw fixation does not compensate for interference gap and subsequent micromotion between the dysplasia cup and its bony bed. Therefore this cup should be restricted to primary surgery alone.

## References

[bib-001] McMinn DJ, Daniel J, Ziaee H, Pradhan C (2008). Results of the Birmingham Hip Resurfacing dysplasia component in severe acetabular insufficiency: A six- to 9.6-year follow-up. J Bone Joint Surg Br.

[bib-002] Hsu JT, Chang CH, Huang HL, Zobitz ME, Chen WP, Lai KA, An KN (2007). The number of screws, bone quality, and friction coefficient affect acetabular cup stability. Med Eng Phys.

[bib-003] Kim SY, Kim DH, Kim YG, Oh CW, Ihn JC (2006). Early failure of hemispheric hydroxyapatite-coated acetabular cups. Clin Orthop Relat Res.

[bib-004] Cheung KW, Yung SH, Wong KC, Chiu KH (2005). Early failure of smooth hydroxyapatite-coated press-fit acetabular cup-7 years of follow-up. J Arthroplasty.

[bib-005] McBryde CW, Shears E, O’Hara JN, Pynsent PB (2008). Metal-on-metal hip resurfacing in developmental dysplasia: A case-control study. J Bone Joint Surg Br.

[bib-006] Nixon M, Taylor G, Sheldon P, Iqbal SJ, Harper W (2007). Does bone quality predict loosening of cemented total hip replacements?. J Bone Joint Surg Br.

[bib-007] Loughead JM, Starks I, Chesney D, Matthews JN, McCaskie AW, Holland JP (2006). Removal of acetabular bone in resurfacing arthroplasty of the hip: a comparison with hybrid total hip arthroplasty. J Bone Joint Surg Br.

[bib-008] Brady OH, Masri BA, Garbuz DS, Duncan CP (1999). Use of reconstruction rings for the management of acetabular bone loss during revision hip surgery. J Am Acad Orthop Surg.

[bib-009] Hsu JT, Chang CH, An KN, Zobitz ME, Phimolsarnti R, Hugate RR, Lai KA (2007). Effects of screw eccentricity on the initial stability of the acetabular cup. Int Orthop.

[bib-010] Perona PG, Lawrence J, Paprosky WG, Patwardhan AG, Sartori M (1992). Acetabular micromotion as a measure of initial implant stability in primary hip arthroplasty. An in vitro comparison of different methods of initial acetabular component fixation. J Arthroplasty.

